# Self-Relevance Attenuates Emotion-Induced Blindness Across Individual, Relational, and Collective Levels

**DOI:** 10.3390/bs16071061

**Published:** 2026-06-26

**Authors:** Tingting Pan, Xinyue Zhang, Xinxin Chen, Jun Wang

**Affiliations:** 1 School of Psychology, Zhejiang Normal University, Jinhua 321004, China; panttt@zjnu.edu.cn (T.P.); xinyue@zjnu.edu.cn (X.Z.); xinxin_chen0104@163.com (X.C.); 2 Zhejiang Philosophy and Social Science Laboratory for the Mental Health and Crisis Intervention of Children and Adolescents, Zhejiang Normal University, Jinhua 321004, China

**Keywords:** emotion-induced blindness (EIB), self-relevance, self-categorization, self-enhancement

## Abstract

Emotion-induced blindness (EIB) refers to the impaired perception of a target due to attentional capture by emotional distractors. While prior research indicates that EIB can be attenuated by enhancing target priority or by regulating emotional distractors through proactive inhibition or passive habituation, it remains unclear whether the intrinsic motivational value of self-relevance can effectively counteract such emotional capture. Grounded in the self-categorization framework, three experiments investigated whether enhancing target self-relevance mitigates EIB across individual, relational, and collective levels. In a two-phase paradigm, neutral images were endowed with high or low self-relevance via associative learning and subsequently presented as targets following emotional distractors in a rapid serial visual presentation (RSVP) task. Results demonstrated that individual (Experiment 1) and relational (Experiment 2) self-relevance significantly reduced EIB, yielding higher accuracy for highly self-relevant targets following negative distractors. For the collective self (Experiment 3), modulation of EIB depended on identity concreteness. Concrete-identity self-relevance (university) effectively buffered against EIB, whereas abstract-identity self-relevance (gender) did not. These findings suggest that target self-relevance may function as a protective buffer that helps reduce emotional interference in EIB across multiple self-levels, potentially offering practical ways to enhance psychological resilience during adverse experiences in real-world settings.

## 1. Introduction

Driven by the robust attentional advantage typically conferred by emotional salience, task-irrelevant emotional distractors often maladaptively disrupt target identification within rapid serial visual presentation (RSVP) streams, a phenomenon termed emotion-induced blindness (EIB; Most et al., 2005). The disruption in target identification stems from the emotional distractor capturing attentional resources, which consequently suppresses the subsequent target within the limited-capacity temporal attention system (Most & Wang, 2011; Wang et al., 2012; Kennedy et al., 2014; Goodhew & Edwards, 2022). This resource competition has been conceptualized through two distinct yet related accounts. The first is the two-stage model, which posits that an emotional distractor occupies the capacity-limited Stage 2 bottleneck at the expense of the target, thereby leaving the target in the transient Stage 1 and causing it to be subsequently lost or overwritten (Chun & Potter, 1995; Goodhew & Edwards, 2022). Similarly, the spatiotemporal competition account suggests that EIB arises when a salient emotional distractor outcompetes the target for limited conscious resources during their spatiotemporal overlap, thereby precluding the target from awareness (Most & Wang, 2011; Wang et al., 2012). Consequently, both accounts attribute EIB to perceptual competition for limited resources, emphasizing a cognitive trade-off between emotional distractors and neutral targets.

Given its potential real-world consequences (e.g., missed sudden traffic hazards due to an emotionally salient billboard), research over the past two decades has increasingly focused on developing approaches to attenuate the EIB effect (e.g., Jia et al., 2022, 2023; Kennedy et al., 2018; Most et al., 2005). These approaches generally fall into two categories based on how they modulate attentional priority, namely strategies aiming to reduce the impact of emotional distractors as well as those seeking to enhance the salience or processing of targets. The first category of approaches focuses on reducing the processing of emotional distractors through either proactive suppression or passive habituation. Research shows that providing explicit distractor information (e.g., categories of emotional images; spatial locations) can significantly reduce their attentional prioritization and improve target perception (Singh et al., 2025; J. Xu et al., 2025; Chan & Saunders, 2025; Kennedy et al., 2018). Regarding the latter mechanism, the recent study shows that effective attenuation is observed through passive habituation to emotional distractors (Jia et al., 2022). In contrast to these approaches on distractors, the second strategy aims to enhance the attentional prioritization of targets in RSVP streams. This involves informing participants of specific target-defining information (i.e., that targets are always rotated buildings) to effectively increase the attentional weight assigned to targets (Most et al., 2005, 2006). Moreover, reward-driven motivation fortifies target processing against emotional interference as high-reward targets elicit a smaller EIB effect than low-reward ones (Yokoyama et al., 2015).

While previous studies have shown that extrinsic incentives (e.g., monetary rewards) or explicit top-down instructions can fortify target processing against emotional distractors, these strategies rely on temporary task demands. In contrast, the present study aimed to examine whether enhancing the intrinsic psychological significance of the target through self-relevance could offer an alternative means to reduce the EIB effect. Unlike monetary rewards, self-relevance engages a chronic, biologically ingrained motivational system toward maintaining self-positivity and stability (Alicke & Sedikides, 2009). Research suggests that it facilitates the processing of associated stimuli, conferring robust advantages in attentional competition. Specifically, regarding its impact on attention, self-related information (e.g., one’s own name, face, or voice) automatically captures attention, yielding faster and more accurate responses compared to other-related information across various visual tasks (Kirk & Cunningham, 2025; Nijhof et al., 2022; Li et al., 2022; Gao et al., 2018; Ocampo & Kahan, 2016; Alexopoulos et al., 2012; Turk et al., 2011; Keyes et al., 2010). According to the self-attention network framework, these advantages are underpinned by an automatic interaction between attention and self-representation mechanisms, which enhances the prioritization of self-relevant stimuli during cognitive processing (Sui & Rotshtein, 2019; Humphreys & Sui, 2016). Moreover, this self-prioritization extends beyond attention to profoundly influence the affective domain. The self-enhancement account posits a motivational drive to discount negative information, thereby shielding the self-concept from threats and sustaining a positive self-view (Alicke & Sedikides, 2009; Sedikides & Strube, 1997). Empirical support for this includes active suppression of negative self-relevance, resulting in worse performance on negative than positive self-targets (Lee et al., 2023; Hu et al., 2020) and a tendency for individuals to perceive negative self-outcomes as less predictable (Mark et al., 2003). Additionally, self-concept priming reduces sensitivity to negative feedback, as indexed by diminished FRN responses (M. Xu et al., 2021). Given its dual functional benefits in facilitating attentional processing and protective buffering against negative interference, self-relevance presents an ideal candidate for reducing the EIB effect.

More importantly, personally meaningful information extends beyond the individual level to interpersonal and collective identities. This aligns with the tripartite self-categorization framework (Turner et al., 1987; Aron et al., 1991; Brewer & Gardner, 1996; Nehrlich et al., 2019), which distinguishes between the individual (personal), relational (interpersonal), and collective (social group) levels. Mirroring the effect observed at the individual level, the relational and collective selves also benefit from prioritized processing. At the relational level, attentional and memory biases favor both established and newly formed social associations. For example, perceptual matching tasks reveal robust we-prioritization, characterized by faster responses to labels and shapes associated with close relationships or new ingroups versus strangers or outgroups (Constable et al., 2019; Enock et al., 2018; Z. Moradi et al., 2015). This advantage extends to memory, as joint action or minimal group assignment enhances recall for partner- or ingroup-associated words over neutral or outgroup stimuli (Zhao et al., 2025; Jeon et al., 2021). At the collective level, research also reveals a pronounced processing advantage for in-group social identities. Specifically, the prioritization of one’s own social identity (i.e., university, family, or sport team) biases selective attention for identity-linked matching stimuli (Enock et al., 2018; Z. Z. Moradi et al., 2017) and facilitates superior memory recall for identity-related information (Bennett et al., 2010; Stewart et al., 2007; Johnson et al., 2002). The processing of social identity information often reaches a level of parity with individual self-referential processing (e.g., Enock et al., 2018; Bennett et al., 2010), yet it appears to be restricted to concrete group identities rather than to abstract social categories (Bennett et al., 2010; Johnson et al., 2002). Specifically, smaller, more defined groups provide vivid prototypes and exemplars that serve as potent mental cues, thereby more effectively activating the self-concept to drive these processing advantages (Johnson et al., 2002).

Grounded in the self-categorization framework that distinguishes among individual, relational, and collective selves, the present study investigated whether target self-relevance attenuates the attentional capture typically elicited by emotional distractors. Specifically, we examined whether assigning self-relevance to targets enhances their identification in an EIB paradigm across these three levels in Experiments 1 through 3. All experiments employed the same two-phase structure comprising a self-association learning task followed by an EIB task. In the self-association learning phase, neutral architectural images of houses and skyscrapers acquired high or low self-relevance via associative conditioning before being presented as targets following emotional distractors in an RSVP stream. We predicted that high self-relevance would attenuate the EIB effect compared to the low self-relevance condition across individual, relational, and collective levels. Specifically, we expected a robust EIB effect as lower target identification accuracy following negative compared to neutral distractors under low self-relevance conditions. Conversely, this performance was expected to diminish or disappear under high self-relevance conditions, reflecting an attenuated EIB effect. Furthermore, in line with Johnson et al. (2002), we predicted that this self-relevance advantage would be modulated by the concreteness of collective identities. Specifically, we hypothesized that the advantage would be weaker or absent for abstract collective identities (i.e., gender group) compared to concrete ones (i.e., university affiliation), thereby resulting in a more pronounced EIB effect in the former.

## 2. Experiment 1: Individual Self-Relevance on EIB

Experiment 1 investigated whether target self-relevance at the individual level counteracts the attentional capture of emotional distractors, thereby mitigating the EIB effect. We hypothesized that participants would successfully learn the self-associations (high vs. low self-relevance) during the self-association learning phase. Crucially, we predicted that the subsequent individual self-relevance of the target would attenuate the EIB effect, resulting in a reduced EIB effect for high- versus low-relevance targets.

### 2.1. Methods

#### 2.1.1. Participants

A prior power analysis using G*Power (version 3.1.9.7; Faul et al., 2007) indicated that a sample size of *N* = 36 would be required to achieve 0.95 power, assuming an effect size (*f*) of 0.25 and an alpha level of 0.05 for a two-way within-subjects ANOVA. In anticipation of potential attrition due to participant withdrawal or below-chance performance, we recruited 40 native Chinese-speaking college students via advertisements. Two participants were subsequently excluded from the analysis due to below-chance performance (<50% accuracy) in the baseline condition (i.e., neutral distractor condition) of the EIB task. The final sample comprised 38 participants (26 women and 12 men; age range = 18–26 years, *M*_age_ = 21.97, *SD* = 2.31). All participants were right-handed, had normal or corrected-to-normal vision, and reported no history of psychiatric or neurological disorders. Prior to participation, participants provided written informed consent in accordance with the Declaration of Helsinki. The study was approved by the Ethics Committee of Zhejiang Normal University.

#### 2.1.2. Apparatus

Stimulus presentation and data collection were controlled via E-Prime 3.0 software (Psychology Software Tools, Inc., Sharpsburg, PA, USA). Visual stimuli were presented against a uniform gray background on a 21-inch Dell LCD monitor (resolution: 1920 × 1080 pixels; refresh rate: 60 Hz). Participants viewed the display from a distance of approximately 60 cm.

#### 2.1.3. Stimuli

Experiment 1 employed a two-phase paradigm that integrated a self-association learning phase (modeled after traditional reward paradigms; Yokoyama et al., 2015; Raymond & O’Brien, 2009) and an EIB procedure (Most et al., 2005). The stimulus set consisted of 72 emotional-scenario pictures, 84 architectural pictures of houses and skyscrapers, and 15 scrambled pictures, all standardized to a resolution of 359 × 269 pixels.

The 72 emotional scenes were selected from the International Affective Picture System (IAPS; Lang et al., 2001) to serve as emotional distractors in the EIB phase, comprising 36 negative and 36 neutral images^1^. Negative pictures predominantly depicted physical pain, violence, or threatening animals (*M*_arousal_ = 6.11, *SD* = 0.75; *M*_valence_ = 2.83, *SD* = 0.87). In contrast, neutral pictures portrayed affectively benign scenarios involving non-threatening people and animals (*M*_arousal_ = 3.70, *SD* = 0.83; *M*_valence_ = 5.80, *SD* = 0.99).

Additionally, architectural pictures comprised 42 house and 42 skyscraper images sourced from Brady and Oliva (2008). In the self-association learning phase, we utilized a balanced subset of 64 pictures containing 32 from each category. All 84 images were subsequently employed in the EIB phase. Of these, 12 were designated for practice, while the remaining 72 functioned as detection targets for the formal experiment. This target set included all exemplars from the learning phase. Additionally, 15 scrambled pictures, generated by combining visual contexts from house and skyscraper images, were used as filler stimuli.

#### 2.1.4. Procedure

**Figure 1:**
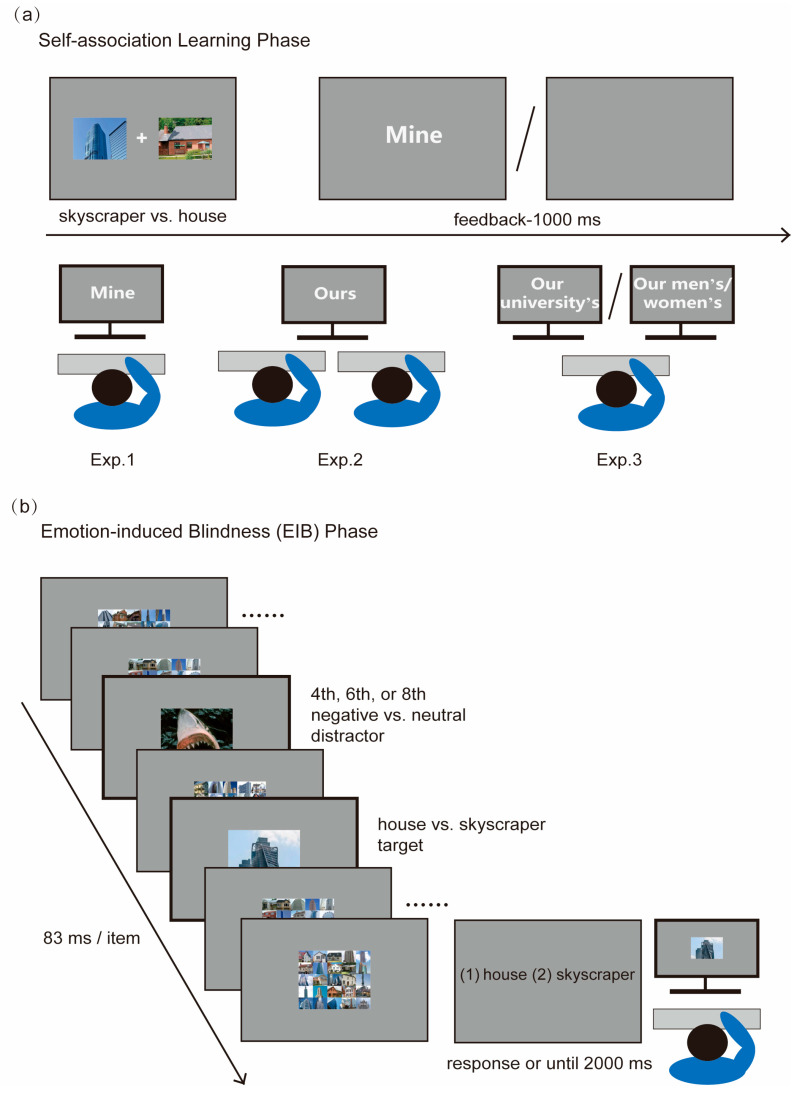
Experimental flowchart. (**a**) the self-association learning phases, where participants learned to associate high and low self-relevance with skyscraper and house images at the individual (Exp.1; “Mine” displayed as “我的”), relational (Exp.2; “Ours” displayed as “我们的”), and collective (Exp.3; “our university’s” displayed as “我们学校的” and “our men’s/women’s” as “我们男/女性的”) levels; (**b**) the emotion-induced blindness (EIB) phase, where participants identified the target category by pressing the “1” key for houses or the “2” key for skyscrapers within an RSVP stream of 17 color pictures.

As illustrated in Figure 1, the experimental procedure was divided into a self-association learning phase and a subsequent EIB task. The former utilized a paradigm adapted from traditional reward-learning tasks (Yokoyama et al., 2015; Raymond & O’Brien, 2009) to establish high or low levels of self-relevance for the stimuli. Subsequently, the EIB task was employed to examine whether target self-relevance could effectively modulate emotional interference.

**Self-association learning phase.** In each trial of Experiment 1, participants first viewed a central white fixation cross (Arial, font size 48) while two colored pictures comprising a house and a skyscraper were simultaneously presented at an eccentricity of 6.43° to the left and right of the center. Participants were required to guess which image was self-relevant by pressing the “z” key (left) or “x” key (right). The spatial positions of the categories were counterbalanced and randomized across trials. Each selection was followed by immediate feedback where the prompt “我的” (“Mine”) appeared for 1000 ms if the choice yielded a self-relevance outcome or a blank screen was shown otherwise. Crucially, the self-relevance outcomes were governed by a probabilistic contingency in which one stimulus category was assigned a high self-relevance probability of 0.8 while the other was assigned a low self-relevance probability of 0.2. The assignment of stimulus categories to these probability levels was counterbalanced across participants. The phase consisted of eight blocks of 64 trials each, with a minimum 20-s rest period provided between blocks.

**Emotion-induced blindness (EIB) phase.** Following the self-association learning phase and a 2-min mandatory break, participants completed the EIB task. Each EIB trial consisted of an RSVP stream of 17 color pictures, where each image was displayed for 83 ms at the center of the screen. Each stream contained one emotional distractor (negative or neutral), one architectural target (house or skyscraper), and 15 scrambled fillers. The emotional distractor was randomly presented at the 4th, 6th, or 8th temporal position in the stream while the target consistently appeared two positions later at Lag 2. Participants were tasked with identifying the target category by pressing the “1” key for houses or the “2” key for skyscrapers. Responses were collected until a key was pressed or a 2000-ms timeout was reached. The EIB phase comprised 144 formal trials, divided into three blocks of 48 trials each. The experiment followed a 2 (distractor valence: negative vs. neutral) × 2 (target self-relevance: high vs. low) within-subjects design. There were 12 trials per condition per block, which were evenly distributed across the three distractor positions and presented in a randomized sequence. Before the formal task, 12 practice trials with immediate feedback were provided to ensure participants were familiarized with the procedure. The entire experimental session lasted approximately 25 to 30 min.

#### 2.1.5. Data Analysis

To quantify the self-association learning process, we calculated the proportion of trials in which participants selected the stimulus assigned a high probability of self-relevance within each block. Consistent with the analysis of previous studies (Yokoyama et al., 2015; Raymond & O’Brien, 2009), a one-sample ANOVA was then conducted on these choice proportions across blocks, with Bonferroni-corrected post hoc tests employed for comparisons between the initial Block 1 and the final Block 8. Furthermore, Spearman’s rank correlation analysis was conducted to assess the statistical significance of the monotonic learning trend.

To investigate whether the individual self-relevance of the target attenuates the EIB effect, the mean recognition accuracy was computed as a function of target self-relevance (high vs. low) and distractor valence (neutral vs. negative) for each participant. Subsequently, we conducted a 2 × 2 repeated-measures ANOVA with target self-relevance and distractor valence as within-subjects factors. All analyses were conducted in JASP (Version 0.95.4) and reported both Frequentist *p*-values and Bayesian Factors to provide a comprehensive evaluation of the statistical evidence.

### 2.2. Results

#### 2.2.1. Results of the Self-Association Learning Phase

In the self-association learning phase, a significant main effect of block emerged for the choice proportion of high self-relevance stimuli (*F* (7, 259) = 8.68, *p* < 0.001, η^2^_p_ = 0.19, *BF*_incl_ = 7.94 × 10^+6^). Follow-up post hoc comparisons indicated that choice proportions were significantly higher in Block 8 than in Block 1 (*t* (37) = 3.75, *p* = 0.017, 95% CI [1.56, 29.19], Cohen’s *d* = 1.09). Furthermore, choice behavior across these learning blocks demonstrated a consistent learning progression, as evidenced by a significant monotonic increase in the preference for high self-relevance stimuli (Spearman *r* = 0.21, *p* < 0.001, 95% CI [0.10, 0.32], Fisher’s *z* = 0.22).

#### 2.2.2. Results of the Emotion-Induced Blindness (EIB) Task

**Figure 2:**
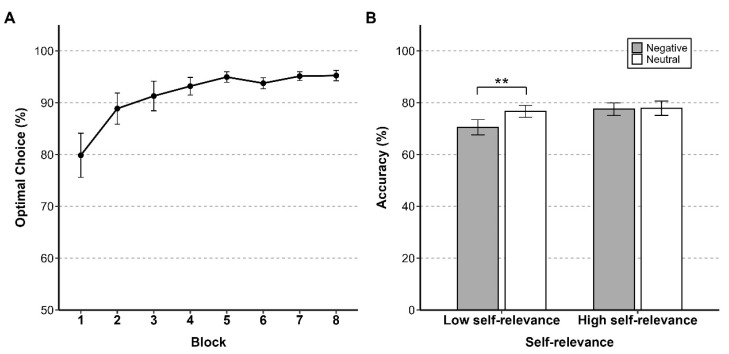
Results of Experiment 1. (**A**) Mean choice proportions for high self-relevance stimuli across blocks during the self-association learning phase. (**B**) Target recognition accuracy in the EIB phase as a function of target self-relevance and distractor valence. Error bars represent ±1 standard error of the mean values (SEM). ** *p* < 0.01.

The two-way repeated-measures ANOVA analysis on recognition accuracy revealed a significant main effect of distractor valence (*F* (1, 37) = 10.46, *p* = 0.003, η^2^_p_ = 0.22, *BF*_incl_ = 2.85), with superior accuracy in the neutral condition compared to the negative condition. No significant main effect of target self-relevance was observed (*F* (1, 37) = 3.44, *p* = 0.072, η^2^_p_ = 0.09, *BF*_incl_ = 1.14). Notably, we observed a significant interaction effect between target self-relevance and distractor valence (*F* (1, 37) = 5.48, *p* = 0.025, η^2^_p_ = 0.13, *BF*_incl_ = 5.46). As illustrated in Figure 2, a follow up simple effect analysis revealed a robust EIB effect in the low self-relevance condition where accuracy was significantly lower for negative distractors (*M* = 70.50%, *SE* = 2.93) compared to neutral distractors (*M* = 76.65%, *SE* = 2.30; *t* (37) = 3.35, *p* = 0.002, 95% CI [2.43, 9.87], Cohen’s *d* = 0.38). In contrast, this EIB effect was abolished in the high self-relevance condition, which showed no significant difference between negative (*M* = 77.47%, *SE* = 2.43) and neutral distractors (*M* = 77.85%, *SE* = 2.77; *t* (37) = 0.29, *p* = 0.774, 95% CI [−2.27, 3.03], Cohen’s *d* = 0.02). Additionally, high self-relevant targets were identified more accurately than low self-relevant targets when faced with negative distractors (*t* (37) = 2.84, *p* = 0.007, 95% CI [2.01, 11.94], Cohen’s *d* = 0.43). This self-relevance advantage was absent in the neutral condition (*t* (37) = 0.46, *p* = 0.648, 95% CI [−4.06, 6.46], Cohen’s *d* = 0.07).

## 3. Experiment 2: Relational Self-Relevance on EIB

Experiment 2 investigated whether target self-relevance at the relational level counteracts the attentional capture of emotional distractors, thereby mitigating the EIB effect. We hypothesized that after successfully learning the self-associations, participants would show a weaker EIB effect for high- to low-relevance targets.

### 3.1. Methods

#### 3.1.1. Participants

The power analysis was identical to Experiment 1. To account for potential data loss, forty-four native Chinese-speaking students were recruited, with six excluded due to below-chance performance (<50% accuracy) in the neutral distractor condition of the EIB task. The final sample comprised 38 participants (34 women and 4 men; age range = 18–25 years, *M*_age_ = 21.03, *SD* = 2.01). All were right-handed, had normal or corrected-to-normal vision, and reported no neuropsychiatric disorders. All participants provided written informed consent. The study was approved by the Ethics Committee of Zhejiang Normal University.

#### 3.1.2. Apparatus, Stimuli, Procedure, and Data Analysis

Experiment 2 adhered to the Experiment 1 protocol but included modifications to the self-association learning phase. Participants were tested in same-sex dyads to establish relational self-relevance. Response keys were assigned to the left (“z”/“x”) and right (“n”/“m”) participants, respectively (“z” and “n” for the left picture and “x” and “m” for the right picture). Mutual correct identifications (i.e., both participants in the dyad responded correctly) were reinforced with the collective prompt “我们的” (“Ours”) for 1000 ms; otherwise, a blank screen was displayed. This dyadic arrangement was restricted to the self-association learning phase. During the subsequent EIB phase, participants performed the RSVP task individually in separate rooms, such that behavioral responses in the EIB task were collected independently for each participant. Data analysis was consistent with that in Experiment 1, and each participant was treated as an independent unit of analysis.

### 3.2. Results

#### 3.2.1. Results of the Self-Association Learning Phase

The self-association learning phase yielded a robust main effect of block on the choice proportion of high self-relevance stimuli (*F* (7, 259) = 19.36, *p* < 0.001, η^2^_p_ = 0.34, *BF*_incl_ = 6.02 × 10^+17^). Post hoc comparisons revealed that choice proportions were significantly higher in Block 8 than in Block 1 (*t* (37) = 7.49, *p* < 0.001, 95% CI [13.25, 34.86], Cohen’s *d* = 1.56). Furthermore, choice behavior across these learning blocks demonstrated a consistent learning progression, as evidenced by a significant monotonic increase in the preference for high self-relevance stimuli (Spearman *r* = 0.40, *p* < 0.001, 95% CI [0.30, 0.49], Fisher’s *z* = 0.43).

#### 3.2.2. Results of the Emotion-Induced Blindness (EIB) Phase

**Figure 3:**
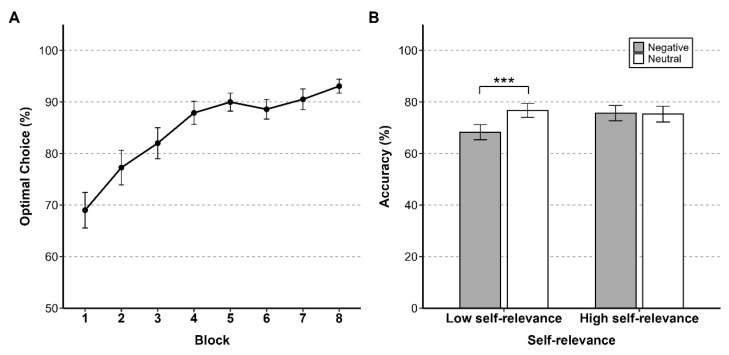
Results of Experiment 2. (**A**) Mean choice proportions for high self-relevance stimuli across blocks during the self-association learning phase. (**B**) Target recognition accuracy in the EIB phase as a function of target self-relevance and distractor valence. Error bars represent ±1 standard error of the mean values (SEM). *** *p* < 0.001.

The two-way repeated-measures ANOVA analysis on recognition accuracy revealed a significant main effect of distractor valence (*F* (1, 37) = 16.00, *p* < 0.001, η^2^_p_ = 0.30, *BF*_incl_ = 8.14), with higher accuracy in the neutral condition than in the negative condition. The main effect of target self-relevance was not significant (*F* (1, 37) = 2.10, *p* = 0.155, η^2^_p_ = 0.05, *BF*_incl_ = 0.60). Notably, we observed a significant interaction effect between target self-relevance and distractor valence (*F* (1, 37) = 13.47, *p* < 0.001, η^2^_p_ = 0.27, *BF*_incl_ = 321.25). As illustrated in Figure 3, a follow-up simple effect analysis revealed a robust EIB effect in the low self-relevance condition, where accuracy was significantly lower for negative distractors (*M* = 68.25%, *SE* = 2.92) than neutral distractors (*M* = 76.73%, *SE* = 2.70; *t* (37) = 5.51, *p* < 0.001, 95% CI [5.36, 11.59], Cohen’s *d* = 0.47). In contrast, this EIB effect disappeared in the high self-relevance condition, with no significant difference between negative (*M* = 75.64%, *SE* = 2.95) and neutral distractors (*M* = 75.26%, *SE* = 3.07; *t* (37) = 0.24, *p* = 0.812, 95% CI [−2.88, 3.65], Cohen’s *d* = 0.02). Moreover, a self-relevance advantage emerged in the presence of negative distractors, where high self-relevant targets were identified more accurately than low self-relevant targets (*t* (37) = 2.90, *p* = 0.006, 95% CI [2.23, 12.56], Cohen’s *d* = 0.41), whereas no such advantage was observed with neutral distractors (*t* (37) = 0.67, *p* = 0.505, 95% CI [−2.95, 5.89], Cohen’s *d* = 0.08).

## 4. Experiment 3: Collective Self-Relevance on EIB

Experiment 3 investigated whether collective self-relevance attenuates the EIB effect and whether the concreteness of social identities, specifically abstract gender and concrete university affiliation, influences this advantage. We predicted that the modulation of self-relevance would vary with identity concreteness, resulting in a weaker EIB effect for the abstract than for the concrete identity.

### 4.1. Methods

#### 4.1.1. Participants

The power analysis was identical to that in Experiment 1. To account for potential data loss, participants were recruited into two distinct collective self groups based on identity concreteness, specifically a gender group for the abstract collective self (*N* = 40) and a university group for the concrete collective self (*N* = 39). Three participants were excluded due to below-chance performance (<50% accuracy) in the neutral distractor condition of the EIB task, including two from the gender group and one from the university group. This resulted in a final sample of 38 participants (19 women and 19 men; age range = 19–25 years, *M_age_* = 21.34, *SD* = 1.94) in the gender group and 38 (21 women and 17 men; age range = 19–28 years, *M_age_* = 21.37, *SD* = 2.38) in the university group. All participants were right-handed, had normal or corrected-to-normal vision, reported no neuropsychiatric disorders, and provided written informed consent. The study was approved by the Ethics Committee of Zhejiang Normal University.

#### 4.1.2. Apparatus, Stimuli, and Procedure

Experiment 3 followed the same protocol as Experiment 1 but included modifications in the self-association learning phase to manipulate the concreteness of collective self-relevance. Participants were assigned to either a gender identity group, characterized by extensive, abstract group reference information, or a university identity group, characterized by more limited, concrete group reference information. In the self-association learning task, participants identified which of two neutral images (e.g., a house or a skyscraper) was associated with their collective self. Correct identifications were reinforced with group-specific feedback. Participants in the gender group received gender congruent prompts (i.e., “我们女性的” [“Our women’s”] for female participants and “我们男性的” [“Our men’s”] for male participants), whereas those in the university group received the prompt “我们大学的” (“Our university’s”). To validate the intended manipulation of concreteness, we adapted the procedure from Johnson et al. (2002). At the conclusion of the experiment, participants provided verbal reports by stating the first thought that came to mind upon seeing the prompt phrases. Additionally, they rated the perceived concreteness of the prompts on a 7-point Likert scale (1 = highly abstract, 7 = highly concrete).

#### 4.1.3. Data Analysis

To validate the concreteness manipulation, we first compared the gender and university groups in terms of the number of concrete items generated and their subjective concreteness ratings. The data analysis for the self-association learning task was identical to that employed in Experiment 1. For the EIB phase, recognition accuracy was analyzed using a 2 × 2 × 2 mixed-design ANOVA, with target self-relevance (high vs. low) and distractor valence (negative vs. neutral) as within-subjects factors, and group (gender vs. university) as the between-subjects factor.

### 4.2. Results

#### 4.2.1. Validation of Concreteness Differences Across Group Identities

Manipulation checks revealed a significant difference in group concreteness between the two groups. Specifically, the university group generated a higher number of concrete items (*χ*^2^(1, *N* = 76) *=* 48.57, *p* < 0.001, Phi-coefficient = 0.80, *BF*_10_ = 2.85 × 10^+11^) and reported significantly higher subjective concreteness ratings compared to the gender group (*M*_gender_ = 2.84, *SE* = 0.16; *M*_university_ = 5.16, *SE* = 0.16; *t* (74) = 10.23, *p* < 0.001, 95% CI [1.86, 2.77], Cohen’s *d* = 2.35, *BF*_10_ = 4.96 × 10^+12^). These results confirm that the collective self associated with university identity is represented more concretely than that associated with gender identity.

#### 4.2.2. Results of the Self-Association Learning Phase

The self-association learning phase yielded a robust main effect of block on the choice proportion of high self-relevance stimuli in both the gender group (*F* (7, 259) = 13.81, *p* < 0.001, η^2^_p_ = 0.27, *BF*_incl_ = 1.89 × 10^+12^) and the university group (*F* (7, 259) = 10.63, *p* < 0.001, η^2^_p_ = 0.22, *BF*_incl_ = 1.23 × 10^+9^). Post hoc comparisons indicated that the proportion of high self-relevance choices was significantly higher in Block 8 than in Block 1 for both groups (gender group: *t* (37) = 4.57, *p* = 0.001, 95% CI [3.87, 25.57], Cohen’s *d* = 1.10; university group: *t* (37) = 3.77, *p* = 0.016, 95% CI [0.90, 15.96], Cohen’s *d* = 1.27). Furthermore, choice behavior across these learning blocks demonstrated a consistent learning progression, as evidenced by a significant increase in the preference for high self-relevance stimuli for the gender group (Spearman *r* = 0.23, *p* < 0.001, 95% CI [0.12, 0.34], Fisher’s *z* = 0.24) and the university group (Spearman *r* = 0.21, *p* < 0.001, 95% CI [0.11, 0.32], Fisher’s *z* = 0.21).

#### 4.2.3. Results of the Emotion-Induced Blindness (EIB) Phase

**Figure 4:**
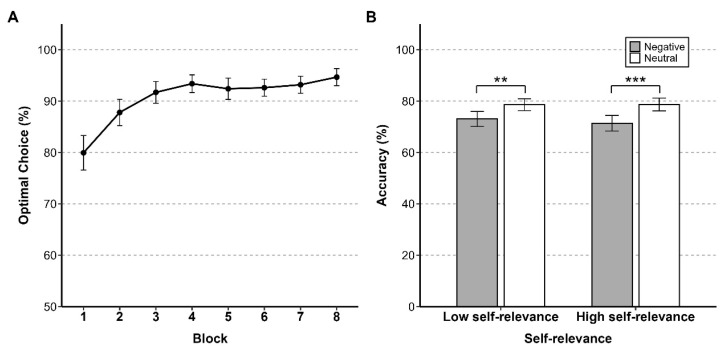
Results of Experiment 3 in the gender group. (**A**) Mean choice proportions for high self-relevance stimuli across blocks during the self-association learning phase. (**B**) Target recognition accuracy in the EIB phase as a function of target self-relevance and distractor valence. Error bars represent ±1 standard error of the mean values (SEM). ** *p* < 0.01, *** *p* < 0.001.

**Figure 5:**
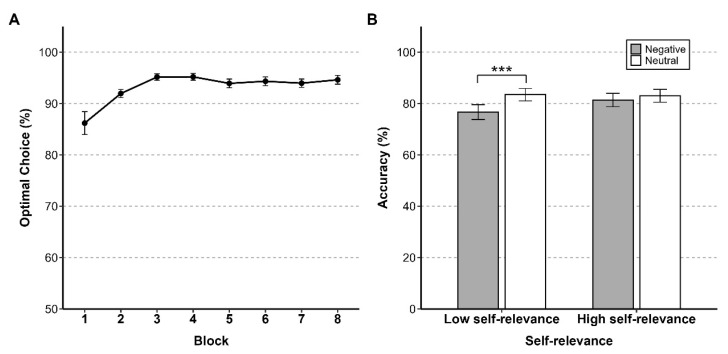
Results of Experiment 3 in the university group. (**A**) Mean choice proportions for high self-relevance stimuli across blocks during the self-association learning phase. (**B**) Target recognition accuracy in the EIB phase as a function of target self-relevance and distractor valence. Error bars represent ±1 standard error of the mean values (SEM). *** *p* < 0.001.

The three-way repeated-measures ANOVA revealed a significant main effect of distractor valence on recognition accuracy (*F* (1, 74) = 55.18, *p* < 0.001, η^2^_p_ = 0.43, *BF*_incl_ = 2.57 × 10^+6^), with higher accuracy in the neutral condition relative to the negative condition. Crucially, a significant three-way interaction among target self-relevance, distractor valence, and group was observed (*F* (1, 74) = 4.86, *p* = 0.031, η^2^_p_ = 0.06, *BF*_incl_ = 3.23). This interaction suggests that the self-relevance advantage on EIB varied between the gender and university groups. No other main effects or interactions reached significance (*F*s (1, 74) ≤ 2.87, *p*s ≥ 0.095, η^2^_p_s ≤ 0.04, *BF*_incl_ values ≤ 1.91). To decompose the three-way interaction, separate 2 (target self-relevance: high vs. low) × 2 (distractor valence: neutral vs. negative) repeated-measures ANOVAs were conducted for each group (see Figure 4 and Figure 5). For the gender group, the analysis yielded only a significant main effect of distractor valence (*F* (1, 37) = 40.34, *p* < 0.001, η^2^_p_ = 0.52, *BF*_incl_ = 4.77 × 10^+3^), with higher accuracy in the neutral condition than in the negative condition. Neither the main effect of target self-relevance (*F* (1, 37) = 0.15, *p* = 0.698, η^2^_p_ = 0.004, *BF*_incl_ = 0.31) nor the interaction effect (*F* (1, 37) = 0.56, *p* = 0.461, η^2^_p_ = 0.01, *BF*_incl_ = 0.33) reached significance. These results indicate that in the gender group, negative distractors consistently impaired recognition performance regardless of the target’s self-relevance level. In contrast, for the university group, a significant main effect of distractor valence was observed (*F* (1, 37) = 17.29, *p* < 0.001, η^2^_p_ = 0.32, *BF*_incl_ = 32.65), whereas the main effect of target self-relevance was not significant (*F* (1, 37) = 1.80, *p* = 0.188, η^2^_p_ = 0.05, *BF*_incl_ = 0.59). Crucially, a significant two-way interaction between target self-relevance and distractor valence emerged (*F* (1, 37) = 6.12, *p* = 0.018, η^2^_p_ = 0.14, *BF*_incl_ = 4.82). Simple effects analysis showed that in the low self-relevance condition, recognition accuracy was significantly higher for the neutral (*M* = 83.45%, *SE* = 2.43) than for the negative distractors (*M* = 76.66%, *SE* = 2.88; *t* (37) = 4.69, *p* < 0.001, 95% CI [3.86, 9.72], Cohen’s *d* = 0.42), demonstrating a robust EIB effect. This EIB effect was eliminated for high self-relevance targets, with no significant difference between negative (*M* = 81.35%, *SE* = 2.61) and neutral distractors (*M* = 82.99%, *SE* = 2.50; *t* (37) = 1.12, *p* = 0.268, 95% CI [−1.32, 4.60], Cohen’s *d* = 0.10). Moreover, a self-relevance advantage emerged in the presence of negative distractors, where high self-relevant targets were identified more accurately than low self-relevant targets (*t* (37) = 2.76, *p* = 0.009, 95% CI [1.24, 8.14], Cohen’s *d* = 0.29), whereas no such advantage was observed in the neutral condition (*t* (37) = 0.22, *p* = 0.825, 95% CI [−3.71, 4.63], Cohen’s *d* = 0.03).

## 5. Cross-Experimental Comparison of Self-Relevance Effects at the Individual, Relational, and Collective Levels

As an exploratory cross-experimental analysis, to evaluate the self-relevance effect on EIB performance across Experiments 1, 2, and 3, we performed a 2 (target self-relevance: high vs. low) × 2 (distractor valence: negative vs. neutral) × 4 (group: individual, relational, gender, and university) mixed-design ANOVA. The analysis revealed significant main effects of distractor valence (*F* (1, 148) = 78.68, *p* < 0.001, η^2^_p_ = 0.35, *BF*_incl_ = 2.33 × 10^+9^) and target self-relevance (*F* (1, 148) = 4.47, *p* = 0.036, η^2^_p_ = 0.03, *BF*_incl_ = 1.24). The two-way interaction between target self-relevance and distractor valence was also significant (*F* (1, 148) = 15.02, *p* < 0.001, η^2^_p_ = 0.09, *BF*_incl_ = 546.06). Crucially, a significant three-way interaction emerged (*F* (3, 148) = 3.69, *p* = 0.013, η^2^_p_ = 0.07, *BF*_incl_ = 14.21), indicating that the modulation of self-relevance on EIB varied across levels of self-representation.

**Figure 6:**
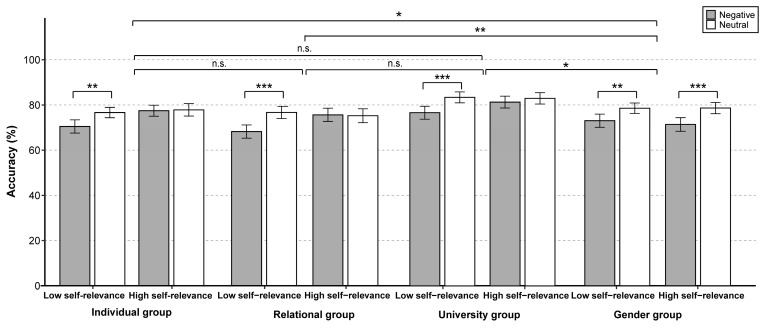
Results of cross-experimental comparison of self-relevance effects. Target recognition accuracy in the EIB phase as a function of target self-relevance and distractor valence across individual, relational, university, and gender groups. Error bars represent ±1 standard error of the mean values (SEM). n.s. = not significant, * *p* < 0.05, ** *p* < 0.01, *** *p* < 0.001.

As shown in Figure 6, to further decompose the three-way interaction and specifically examine whether the modulation of self-relevance on EIB was comparable across the individual, relational, and university groups, we first conducted a 2 (target self-relevance) × 2 (distractor valence) × 3 (group: individual, relational, university) mixed-design ANOVA. This analysis revealed no significant three-way interaction (*F* (2, 111) = 0.73, *p* = 0.484, η^2^_p_ = 0.01, *BF*_incl_ = 0.06), suggesting that the effect of self-relevance on EIB did not differ significantly among these three levels of self-representation in this exploratory cross-experimental comparison. Subsequently, separate 2 × 2 × 2 mixed-design ANOVAs were conducted to compare the gender group with the individual and relational groups. Significant three-way interactions emerged in both comparisons (gender vs. individual: *F* (1, 74) = 4.90, *p* = 0.030, η^2^_p_ = 0.06, *BF*_incl_ = 6.47; gender vs. relational: *F* (1, 74) = 9.98, *p* = 0.002, η^2^_p_ = 0.12, *BF*_incl_ = 41.83). These results align with the pattern observed between the gender and university groups in Experiment 3, preliminarily indicating that the modulation of self-relevance on EIB was stronger at the individual and relational levels than at the gender-based collective level.

## 6. Discussion

The present study investigated whether the self-relevance of target stimuli could attenuate the EIB effect. To address this, we employed a two-phase paradigm integrating a self-association learning phase (Yokoyama et al., 2015) with an EIB procedure (Most et al., 2005). By pairing neutral targets with self-relevant prompts during the learning phase, we systematically investigated the manifestations of the self-relevance advantage across the individual (Experiment 1), relational (Experiment 2), and collective (Experiment 3) levels of the self. Consistent with our hypothesis, a significant modulation effect of self-relevance on EIB was observed at both the individual (Experiment 1) and relational (Experiment 2) levels. Specifically, while a robust EIB effect was observed for low self-relevant targets, it was virtually abolished for high self-relevant ones. This pattern was driven primarily by trials with negative distractors, in which high self-relevant targets were identified significantly more accurately than low self-relevant targets. In contrast, no such difference in accuracy was found in trials with neutral distractors. In Experiment 3, however, the self-relevance advantage was found to be contingent upon the concreteness of the social identity. For the concrete collective self (university), self-relevance similarly reduced the EIB effect. In contrast, self-relevance provided no such modulation for the abstract collective self (gender), where a robust EIB effect persisted. Furthermore, exploratory cross-experimental comparisons suggested that the modulation effect of self-relevance was similar in magnitude across the individual, relational, and university groups, yet all were significantly stronger than the effect observed in the gender group.

Extending beyond previous research that relied on temporary and extrinsic manipulations (Jia et al., 2022; Kennedy et al., 2018; Most et al., 2005), our study provides the first evidence that self-relevance functions as a stable, intrinsic target attribute that modulates the perceptual competition underlying EIB. Importantly, the self-relevance advantage is not limited to the individual domain but operates across the full spectrum of the self, as defined by the tripartite model, which includes individual, relational, and collective components (Turner et al., 1987; Brewer & Gardner, 1996; Nehrlich et al., 2019). In line with this model, we observed a comparable attenuation of the EIB effect by self-relevance at the individual (Experiment 1), relational (Experiment 2), and collective (Experiment 3) levels. Furthermore, consistent with prior research, the self-relevance advantage observed in this study lends support to the protective buffering hypothesis, as evidenced by a significant accuracy advantage for high over low self-relevant targets in the presence of negative distractors. This aligns with prior fMRI work showing that self-related processing modulates activity in emotion and attention regions (Herbert et al., 2011b; Fossati et al., 2003). Emotion processing recruits a distributed set of brain regions, including the amygdala and key nodes of the salience network, such as the anterior cingulate cortex and insula (Zhou & Becker, 2025; Duggirala et al., 2022). Importantly, self-relevant social information has been shown to directly modulate activity in the amygdala (Conty & Grèzes, 2012) and the salience network (Perini et al., 2018), before full emotional evaluation occurs (Yankouskaya et al., 2023). In the self-attention network framework (Sui & Rotshtein, 2019), self-relevance is closely intertwined with attentional processing, supported by evidence linking self-relevance to increased neural activation within a specific network involving the vmPFC for self-representation, the pSTS for attention, and the DLPFC for attentional control (Martínez-Pérez et al., 2020; Humphreys & Sui, 2016; Sui et al., 2013). These emotion and attention regions have been consistently implicated in the processing of emotionally salient stimuli (Duggirala et al., 2022; Dolcos et al., 2020; Dixon et al., 2017), particularly within the context of the EIB paradigm (John et al., 2016; Most et al., 2006). Thus, the abolition of the EIB effect under high self-relevance may reflect self-related modulation of emotion–attention networks. Specifically, when negative distractors are strongly linked to the self, self-relevance may reduce the disruptive impact of these stimuli by attenuating activity in regions responsible for detecting and prioritizing emotional salience. An alternative, but complementary, explanation is that self-relevance engages higher-order control mechanisms. Research indicates that self-related processing recruits the default mode network (DMN) (Molnar-Szakacs & Uddin, 2013), which involves the medial prefrontal cortex (mPFC) (Yin et al., 2021; Martínez-Pérez et al., 2020), a region closely linked to the monitoring and regulation of emotional responses (Bermond, 2008; Zhou & Becker, 2025). Evidence from EEG studies further supports this regulatory account, showing that emotional modulation of neural markers, such as the N400 and LPP, is especially pronounced in self-relevant contexts (Zhang et al., 2024; Herbert et al., 2011a, 2011c; Watson et al., 2007). Consistent with this view, self-directed attention has been found to reduce negative emotional processing by increasing activity in prefrontal and parietal regions associated with top-down control, while decreasing activity in regions involved in emotional processing such as the amygdala (Dolcos et al., 2020). Consequently, high self-relevance may weaken the EIB effect by enhancing prefrontal control over emotional distraction, thereby reducing the attentional capture typically produced by negative stimuli. Taken together, these findings suggest that self-relevance enhances target prioritization either by engaging higher-order control mechanisms and/or by directly modulating emotion–attention networks, thereby counteracting the disruptive influence of emotional distractors in the EIB paradigm.

However, in contrast to prior studies suggesting robust attentional prioritization for self-related stimuli (Kirk & Cunningham, 2025; Gao et al., 2018; Keyes et al., 2010), we found no accuracy advantage for high over low self-relevant targets in the neutral distractor condition. This discrepancy likely stems from the differing operationalizations of self-relevance across studies. Previous research has often relied on ecologically valid stimuli (e.g., personal photos, voices, names). These well-established self-cues benefit from extreme familiarity (Devue & Brédart, 2011; Amodeo et al., 2023), are deeply integrated into the long-term self-schema through extensive experience (Conway & Pleydell-Pearce, 2000), and thus command a robust intrinsic attentional priority (Sui & Rotshtein, 2019). In comparison, newly formed self-associations (e.g., with geometric shapes) inherently lack this foundational familiarity and schematic integration. Consistent with this distinction, well-established self-cues reliably elicit stronger self-prioritization effects than newly formed associations across various paradigms. This is evidenced not only by faster responses, higher accuracy, and enhanced P3 amplitudes (Żochowska et al., 2023), but also by their superior ability to capture attention in dot-probe tasks compared to newly associated shapes or pseudowords (Orellana-Corrales et al., 2020, 2021). Thus, the newly associated targets in our experiment likely possessed insufficient potency to generate a broad attentional advantage akin to that of well-established self-representations.

Another notable finding of the present study is that the collective self-relevance advantage was found to be contingent upon the concreteness of the social identity in Experiment 3. The distinction between concrete and abstract collective identities examined here relies on the availability of vivid prototypes and exemplars (Johnson et al., 2002). This was evidenced by the results that the university group generated a significantly higher number of concrete items (e.g., familiar scenes and figures) compared to the gender group. In turn, the availability of vivid prototypes and exemplars influences the activation of social identification (Klimstra et al., 2023; Castelli et al., 2004; Smith & Zarate, 1990), involving the knowledge of belonging to certain social groups alongside the emotional and evaluative significance associated with that membership (Tajfel, 1972; Brewer, 2003). Crucially, social identification can drive and modulate the collective self-relevance effect. Evidence shows that collective self-advantage intensifies with stronger social identification (Liu et al., 2015; K. Xu et al., 2014). Neuroimaging studies support this by revealing that high group identification fosters significant neural overlap between self- and group-perception (Scheepers et al., 2013). Furthermore, akin to the individual self-concept, strong in-group identification motivates individuals to favor positive outcomes and diminish the impact of negative feedback, thereby serving a self-protective function (Hodson et al., 2003; Hansen & Sassenberg, 2006; Tarrant et al., 2012). Accordingly, the asymmetric results in Experiment 3 can be attributed to a concrete in-group identity (e.g., university affiliation) readily triggering strong social identification, thereby attenuating the EIB effect. Conversely, an abstract identity (e.g., gender) lacks vivid prototypes, failing to elicit the same level of identification and its subsequent protective effects.

Several limitations and future directions warrant consideration. First, our newly associated self-targets showed an advantage only against negative distractors, whereas ecologically valid self-cues (e.g., personal names, faces, voices) often elicit broader attentional prioritization (Alexopoulos et al., 2012; Keyes et al., 2010; Nijhof et al., 2022). Future EIB studies should therefore incorporate more naturalistic self-related materials, which better approximate everyday self-referential processing, to determine whether self-prioritization confers broader attentional benefits across emotional contexts. Second, while behaviorally we observed a protective buffering function across multiple self-levels, the underlying neural dynamics remain unclear. Subsequent neuroimaging work is needed to clarify whether shared or distinct neural networks support these self-relevance advantages. Third, the samples across all three experiments consisted entirely of college students. Although using such a homogeneous sample provided a controlled context to test our hypotheses, it restricts the external validity of the findings. Because these results may not fully generalize to the broader population, future research should aim to replicate and extend the current findings using more diverse, non-student samples to establish the robustness and broader applicability of the observed effects.

## 7. Conclusions

This study provides novel evidence that emotional interference in the EIB task can be mitigated by enhancing the intrinsic prioritization of targets through self-relevance. Distinct from prior research, we observed that this self-relevance advantage emerged specifically in the presence of negative distractors, thereby buffering against negative emotional interference. This advantage remained stable across individual and relational levels and extended to the concrete collective self linked to high social identification. Together, these findings highlight the robustness of the self-relevance advantage and establish a stable, intrinsic approach to attenuating EIB, complementing existing strategies that rely on transient or extrinsic manipulations. This further suggests that activating the self-concept could be a promising strategy with potential real-world applicability. For instance, individuals might enhance psychological resilience during adverse experiences by focusing on self-relevant entities, including personal belongings, close relationships, or supportive social identities.

## Data Availability

All data and code can be found at https://osf.io/78x3z/overview (accessed on 5 May 2026).

## Note

1 Negative picture (IAPS codes): 1040, 1050, 1052, 1080, 1090, 1101, 1111, 1120, 1220, 1270, 1280, 1300, 1301, 1930, 2800, 2810, 3030, 3120, 3160, 3170, 3181, 9405, 3230, 3350, 3530, 3550, 6312, 6313, 6350, 6510, 6560, 6571, 9042, 9400, 6550, 3071; Neutral picture (IAPS codes): 1510, 1450, 1560, 1650, 1590, 1600, 1660, 1810, 1620, 1670, 1540, 1812, 1850, 1900, 1910, 1942, 2214, 2240, 2372, 2381, 2383, 2190, 2410, 2490, 2480, 2485, 2495, 2570, 2575, 2580, 2749, 2840, 2870, 2890, 7550, 9210.

## Abbreviations

The following abbreviations are used in this manuscript:

DMN	Default mode network
DLPFC	Dorsolateral prefrontal cortex
EEG	Electroencephalography
fMRI	Functional magnetic resonance imaging
FRN	Feedback-related negativity
mPFC	Medial prefrontal cortex
pSTS	Posterior superior temporal sulcus
vmPFC	ventromedial prefrontal cortex
